# Automated classification of natural habitats using ground-level imagery

**DOI:** 10.1371/journal.pone.0351335

**Published:** 2026-06-17

**Authors:** Mahdis Tourian, Remy Vandaele, Sareh Rowlands, Max Fancourt, Rebecca Mein, Bethany Hadfield, Alison Saunders, Sophie Potter, Amy Woodget, Hywel T. P. Williams

**Affiliations:** 1 Centre for Environmental Intelligence, University of Exeter, Exeter, United Kingdom; 2 Department of Computer Science, Faculty of Environment, Science and Economy, University of Exeter, Exeter, United Kingdom; 3 Natural England, York, United Kingdom; Sultan Qaboos University, OMAN

## Abstract

Accurate classification of terrestrial habitats is critical for biodiversity conservation, ecological monitoring, and land use planning. Several habitat classification schemes are in use, typically based on analysis of satellite imagery and validation by field ecologists. Here, a methodology is presented for classification of habitats based solely on ground-level imagery (photographs), offering improved validation and enhanced ability to classify habitats at scale (e.g., using imagery from citizen science). In collaboration with Natural England, a public sector organisation with responsibility for nature/biodiversity conservation in England, this study develops a classification system that applies deep learning to ground-level habitat photographs, categorising each image into one of 16 distinct classes following the established ‘Living England’ framework. Images were pre-processed using resizing, normalisation, and augmentation techniques, while resampling was used to balance classes in the training data and enhance model robustness. A custom deep learning classifier based on the DeepLabV3-ResNet101 architecture was developed and fine-tuned to assign a habitat class label to ground-level photographs. Using five-fold cross-validation, the model demonstrated strong overall performance across 16 habitat classes, with accuracy and F1-scores varying between classes. This approach supports robust, scalable habitat classification based on balanced and well-prepared training data. Across all folds, the model achieved a mean F1-score of 0.63, with some habitat classes such as Bare Sand (BS) and Coniferous Woodland (CW) reaching values above 0.87. High performance was achieved for visually distinct habitats and lower performance for visually mixed or ambiguous classes. These findings demonstrate the potential of this approach for ecological monitoring. Ground-level imagery is easily obtained and accurate computational methods for habitat classification based on such data have many potential applications. To support use by practitioners, a simple web application is also provided that allows classification of uploaded images using the trained model.

## Introduction

Habitat classification is an essential tool for biodiversity conservation, land management, and ecological monitoring. While natural ecosystems are complex and exhibit many idiosyncrasies, habitat classification frameworks provide a method for reducing this diversity into a small number of defined classes. While a ‘habitat’ is typically defined with respect to a single species, the classes used by such frameworks refer to ‘habitat types’ that support many species and are often defined by the combination of vegetation and other biotic/abiotic factors that support wildlife in a particular location. Each habitat type in a classification framework serves as a proxy indicator for the range of species present at a given location, as well as for key features of the local ecosystem such as soil type, moisture balance, and climatic conditions [1].

Various habitat taxonomies exist. For example, the Phase 1 framework for the UK [2] divides terrestrial habitat types into ten categories (e.g., A: Woodland and scrub, B: Grassland and marsh, C: Tall herb and fen, D: Heathland, and so on). More recently, the Living England framework labels every part of England with one of 16 main habitat classes [3].

Traditional habitat classification in the UK and Europe has long been rooted in vegetation science and phytosociology, where plant species composition is the primary diagnostic tool for defining ecological units. Systems such as the National Vegetation Classification (NVC) [1] and the broader phytosociological tradition stemming from Braun-Blanquet [4,5] group vegetation into communities based on characteristic species assemblages and their relationships to environmental gradients. These frameworks have been foundational for conservation assessment, ecological monitoring, and habitat mapping for decades [6]. Similar approaches underpin European classification schemes such as EUNIS, where vegetation communities form the backbone of higher-level habitat categories [7].

Such community-based methods integrate floristic, structural, and environmental information, allowing ecologists to distinguish between habitats that may appear superficially similar but differ significantly in species composition or ecological function. In contrast, appearance-based approaches relying on imagery alone capture primarily structural and textural cues. Ground-level photographs therefore represent a simplified view of habitat identity, lacking much of the floristic resolution that traditional vegetation surveys provide. The study described here should therefore be understood as complementary: it leverages visual signatures that can be captured at scale, while not replacing species-based classification methods that remain essential for fine-grained ecological interpretation.

Applying a given taxonomy using manual classification methods is labour-intensive, time-consuming, and subject to human error [8]. This has led to the adoption of automated techniques based on satellite imagery and remote sensing, enabling scalable, cost-effective, and repeatable habitat mapping across large and heterogeneous landscapes [9]. However, satellite-based approaches still require ground-level validation, typically performed by trained ecologists, which demands substantial time and effort.

This work explores whether ground-level imagery (e.g., photographs of habitats) can be used to automatically classify the habitat type present at a location using computer vision techniques. If successful, this approach offers several potential benefits: ground-level imagery is easily obtained, and accurate automated classification would enable high-resolution, consistent habitat mapping at scale, without requiring expert annotation.

Ground-level photographs primarily capture the visible structural characteristics of habitats, such as vegetation form, canopy cover, surface texture, and broad landscape context. While such imagery may not always encode fine-grained ecological distinctions based on species composition or soil and hydrological conditions, it often contains sufficient visual information to distinguish broad habitat structure. In particular, ground-level imagery captures habitat structure but not the floristic distinctions that underpin many vegetation-based classification systems. In practical ecological workflows, narrowing a site to a small set of plausible habitat types can already provide valuable decision support, allowing automated predictions to be combined with contextual knowledge, site metadata, or expert judgement. This suggests that ground-level imagery may be particularly well suited to supporting habitat classification as part of a hybrid human–machine workflow, rather than as a fully autonomous replacement for ecological assessment.

Recent advancements in deep learning [10], particularly in convolutional neural networks (CNNs), have made image-based ecological analysis increasingly feasible. Deep learning methods are already playing a growing role in ecological monitoring and ecosystem science, supporting tasks such as wildlife detection from camera traps, acoustic species recognition, and environmental change monitoring from satellite imagery [11,12]. These tools are also advancing toward greater interpretability and causal understanding through hybrid models and attention mechanisms.

Numerous studies have explored computer vision methods for wildlife monitoring, and the field continues to evolve rapidly. For example, Miao et al. [13] used CNNs to classify African wildlife in camera-trap images, achieving high accuracy and employing interpretability techniques such as Grad-CAM (Gradient-weighted Class Activation Mapping) [14] to highlight image regions influencing model predictions. Similarly, Otsuka et al. [15] applied deep learning to classify seabird behaviours using accelerometer data, demonstrating the effectiveness of hybrid CNN–LSTM models combined with self-attention mechanisms. Bi et al. [16] showed how deep learning can enhance ecosystem health assessments by integrating complex environmental datasets efficiently.

With regard to the specific problem of habitat type classification, recent advances have been made, though key challenges remain. In the marine domain, Game et al. [17] proposed a hybrid CNN–SVM model to classify benthic habitats using imagery. Their results demonstrated the potential for simplified and practical deep learning pipelines in ecological research. Leblanc et al. [18] developed a method to classify terrestrial habitat types using the EUNIS framework. Rather than relying on imagery, their approach combined species composition data with environmental variables in a deep learning model to classify vegetation across Europe. The study underscored the importance of integrating biological and environmental data for robust ecological predictions. However, such detailed datasets are not always available or complete at national scales like the UK, particularly for fine-grained habitat mapping. This limitation motivates an exploration of whether ground-level imagery alone can support reliable habitat classification through computer vision.

Meanwhile, habitat classification approaches using satellite imagery are more common in terrestrial contexts. For instance, the Living England project [3] uses satellite data combined with machine learning algorithms to segment the landscape into polygons and assigns each polygon to one of 16 single-type habitat classes. The approach emphasises national-scale consistency and is updated regularly to support monitoring and land-use decision-making. Similarly, the Centre for Ecology & Hydrology (CEH) Land Cover Maps [19] provide long-term, high-resolution classifications of UK land cover based on satellite imagery, with supervised classification techniques applied to multispectral data. These frameworks demonstrate the scalability and repeatability of satellite-based approaches, though they still require field validation and may struggle with fine-grained habitat type distinctions, especially in heterogeneous or transitional landscapes.

To the best of current knowledge, no peer-reviewed studies have yet examined the use of ground-level imagery for terrestrial habitat classification. However, a recent unpublished preprint (arXiv:2507.04017) [20] and a new commercial offering from Mozaic Earth [21] indicate growing interest in this application area and the need for rigorous peer-reviewed study. Indeed, ground-level images can be easily acquired and shared by non-experts using devices such as smartphones, for example through social media scraping or citizen science initiatives. These images could then be classified by experts or algorithms to provide ground-level habitat information. Image-based habitat classification faces several challenges. Previous image-based ecological studies have reported frequent misclassifications in cases involving complex or visually similar habitats [22], and noted difficulties in generalising across geographic regions [23]. Ethical and technical challenges such as data bias, interpretability, and model transferability must also be addressed [11,16].

Against this background, this study evaluates the use of ground-level habitat photographs for image-based habitat classification within a national ecological framework. Using images collected by professional ecologists and labelled according to the Living England taxonomy, the work examines the extent to which standard deep learning methods can classify habitats based on visual structure alone, rather than the full set of biotic and abiotic criteria used in traditional habitat definitions, and how classification performance differs between visually distinct habitat types and visually ambiguous or transitional classes. The analysis further considers the ecological and practical implications of observed misclassification patterns for habitat monitoring and decision-making, and assesses under what conditions ground-level imagery might complement existing satellite-based habitat mapping approaches, as well as the main limitations in terms of generalisability.

## Methods

The experiment created a neural network classifier to automatically label images of habitats with a class from the Living England habitat classification scheme. This is a challenging computer vision task since a ‘habitat’ is an assemblage of co-occurring vegetation and biotic/abiotic factors rather than a single object. While habitat types can be visually distinctive, the classification depends on the simultaneous presence of multiple visual features including vegetation, colour, bare rocks/soil, etc. The methodology is based on transfer learning with pre-trained vision models that have demonstrated strong performance in object classification. This section describes the training dataset of labelled habitat images, how the dataset was prepared for use, the transfer learning approach and the pre-trained vision models used, and the experiment design and performance metrics.

### Dataset

The dataset consists of 41,499 RGB-encoded ground-level photographs captured by ecologists working for Natural England as part of the Living England habitat mapping initiative [24]. Each image was taken by an ecologist during a field site visit, labelled with a habitat class from the Living England classification scheme, and linked with metadata on field site, location, and collection date. To characterise temporal coverage, S1 Fig summarises the distribution of the available image-date metadata by habitat class across year-month periods, and S2 Fig provides a seasonal summary. These metadata were used only for descriptive purposes and were not included as model inputs in the present study.

In addition to the number of images per habitat class, Table 1 reports the number of unique recorded locations for each class, defined as unique (X, Y) coordinate pairs in the metadata. These metadata were used only for descriptive purposes and were not included as inputs to the model.

**Table 1:** Number of images and unique locations per habitat category in the Living England dataset. A unique location was defined as a unique (*X*,*Y*) coordinate pair in the metadata. Location metadata were used only to describe dataset composition and were not used as model inputs.

Category	Abbr.	Images	Unique
Arable and Horticultural	AH	2359	1538
Bare Sand	BS	957	523
Bare Ground	BG	1018	599
Bog	BOG	1750	909
Bracken	BRA	2567	1451
Broadleaved, Mixed and Yew Woodland	BMYW	3187	1785
Built up areas and Gardens	BUAG	754	464
Coastal Saltmarsh	CS	1008	606
Coastal Sand Dunes	CSD	1546	777
Coniferous Woodland	CW	371	206
Dwarf Shrub Heath	DSH	4699	2403
Fen, marsh and swamp	FMS	2044	1235
Improved and Semi-Improved Grassland	IG	10555	5745
Scrub	SCR	2053	1151
Unimproved grassland	UG	6172	3137
Water	WAT	459	314
**Total**		**41499**	**22841**

Living England is a national-scale habitat-mapping project covering the entirety of England up to Mean High Water Springs. It was first launched in 2016 as Defra’s “Living Maps” pilot and has since evolved into its current baseline 2022–23 iteration, produced from 2022–23 data and published in September 2024 [25,26]. It maps 16 broad habitat classes aligned with UK Biodiversity Action Plan categories, plus solar farms. A hybrid workflow uses segmentation based on a combination of Sentinel-2 seasonal image mosaics, LiDAR-derived canopy height models, focal texture layers, and OS MasterMap topography (urban and water features), implemented in eCognition (Trimble) software, and applies object-based random forest classification alongside targeted field surveys for model training and validation. The current version achieves approximately 87% overall accuracy and is intended for biennial updates under an Open Government Licence.

Table 1 shows the number of images available for each habitat class. The dataset is highly imbalanced, with certain categories such as *Improved and Semi-Improved Grassland* (IG) having over 10,000 images, while others like *Coniferous Woodland* (CW) contain fewer than 400 images. Class imbalance is known to impact classification performance in computer vision and was addressed during model training through resampling (see below).

**Fig 1:**
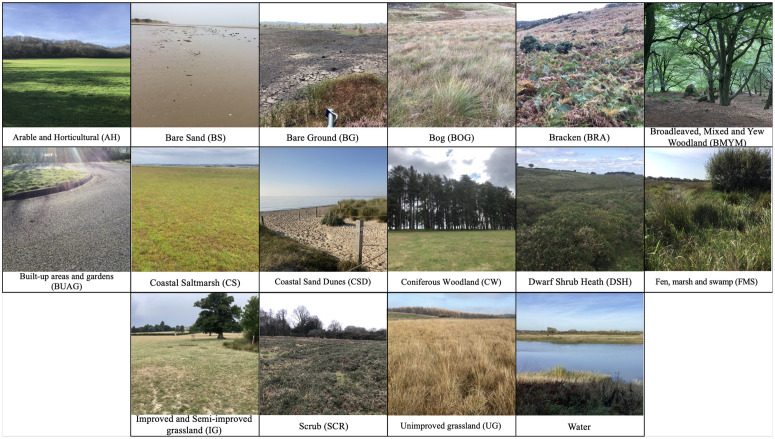
Examples of the 16 habitat classes in the Natural England habitat classification scheme. Images contain or are derived from Living England survey data © Natural England 2024, licensed under the Open Government Licence v3.0. Additional imagery sources include: Copernicus Sentinel data © 2022–2023 (processed by the authors) and APGB aerial imagery accessed under institutional licence (subject to the APGB End User Licence).

Examples of images from each class are shown in Fig 1.

### Classifier model & training

A transfer learning approach was adopted by leveraging pre-trained deep convolutional neural networks (CNNs), which were fine-tuned for the habitat classification task using ecologist-captured imagery. In preliminary work, a standard image classification model based on Inception-ResNet-v2 and InceptionV3 was tested for the habitat classification task. These models were trained for 200 epochs with a custom classification head for habitat prediction. Both models exhibited early convergence, with a peak validation accuracy of 55% and 44% and high variance across epochs, indicating instability and limited learning capacity. A classifier based on DeepLabV3, pre-trained on outdoor scene segmentation and better suited to the habitat image dataset, was then evaluated. This model showed better performance and was chosen for further development.

The final model was based on DeepLabV3 with a ResNet-101 backbone, originally designed for semantic segmentation [27]. DeepLabV3 incorporates atrous convolutions and an Atrous Spatial Pyramid Pooling (ASPP) module to capture multi-scale context efficiently. The architecture was adapted for image-level classification by replacing the segmentation head with a 1×1 convolutional layer followed by global average pooling and flattening. This design leverages the spatially aware, high-resolution features of DeepLabV3 while outputting image-level predictions. Dropout (rate = 0.5) was applied for regularization.

### Baseline architectures and model selection

Before adopting the DeepLabV3-ResNet101 architecture, several standard image-classification models were evaluated as alternative architectures. As an example, an Inception-ResNet-v2 classifier pre-trained on ImageNet was fine-tuned for the habitat classification task. This classifier consisted of the pre-trained convolutional network followed by global average pooling and a fully connected layer with 16 outputs (corresponding to the 16 Living England habitat classes) using a SoftMax activation.

The Inception-ResNet model used the same input preprocessing as the final model (RGB images resized and normalised using ImageNet statistics) and the same balanced training set (1,000 images per class, obtained through random subsampling and augmentation). Training used a 90/10 train–validation split, cross-entropy loss, and the AdamW optimiser with a learning rate of 10^−4^ and a weight decay of 10^−4^. Early stopping was applied based on validation accuracy, with a maximum of 200 epochs. Top-*k* accuracy (for *k* = 1 and *k* = 3) and per-class accuracy were recorded.These experiments were exploratory and used a single train–validation split, prior to the adoption of five-fold cross-validation for the final model evaluation described below.

**Fig 2:**
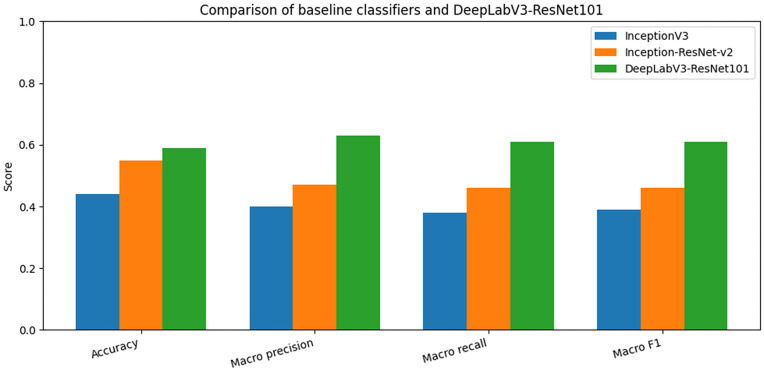
Comparison of performance metrics for InceptionV3, Inception-ResNet-v2, and DeepLabV3–ResNet101. Metrics shown include accuracy, macro-precision, macro-recall, and macro-F1 score. DeepLabV3–ResNet101 consistently achieves higher performance across all metrics.

These preliminary experiments were used to assess whether a generic image classifier was sufficient, or whether a scene-oriented architecture trained on outdoor imagery, such as DeepLabV3, would provide additional benefits for complex habitat imagery. Results (see Fig 2) suggested that the DeepLabV3 architecture performed better, and this architecture was therefore selected for subsequent cross-validated experiments. Lighter-weight architectures may be preferable for deployment scenarios; however, a systematic evaluation of deployment-optimised models was beyond the scope of this study.

### Data preparation

Since the chosen deep learning model, DeepLabV3–ResNet101, was pre-trained on the ImageNet dataset [28], a similar preprocessing scheme was adopted to ensure compatibility and effective transfer learning. All images were resized to 224×224 pixels and normalised using standard ImageNet statistics (mean = [0.485, 0.456, 0.406]; standard deviation = [0.229, 0.224, 0.225]). For the training set, data augmentation techniques were applied, including random horizontal flipping, ±15° rotation, colour jittering, and the AutoAugment ImageNet policy [29], to improve generalisation and mitigate overfitting. Validation images were resized and normalised without augmentation. No explicit segmentation or foreground-object removal was applied during preprocessing. The classifier therefore operated on the full RGB image after resizing, normalisation, and augmentation. To address class imbalance in the Natural England dataset, the number of training images per class was standardised to 1,000. For overrepresented classes, images were randomly subsampled; for underrepresented classes, the dataset was synthetically expanded using the aforementioned augmentation strategy until 1,000 images were reached. This threshold was selected as a trade-off between computational feasibility and representation diversity, and aligns with common practice in image classification pipelines. Training was accelerated using automatic mixed precision (AMP) [30], which reduces memory usage and improves runtime efficiency without compromising model accuracy.

### Experimental setup

The model was trained using the Cross-Entropy Loss function and optimized with AdamW (learning rate = 1e-4, weight decay = 1e-4). Mixed precision training was employed using PyTorch AMP to accelerate training and reduce memory usage [31]. To further minimize GPU memory consumption, gradient checkpointing (using *torch.utils.checkpoint*) was applied to both the backbone and classifier components of the network, allowing recomputation of intermediate activations during backpropagation. Training was conducted with a batch size of 16 for up to 100 epochs, with early stopping triggered if performance did not improve for seven consecutive epochs.

Accuracy (see below) was used as the primary metric to monitor model performance during training. Specifically, validation accuracy was tracked across epochs, and the model with the highest validation accuracy was selected as the best-performing checkpoint for each fold.

To validate the method, five-fold cross-validation was used: the dataset was divided into five folds, and the model was trained and evaluated five times, each time using a different fold as the validation set and the remaining folds for training.

**Fig 3:**
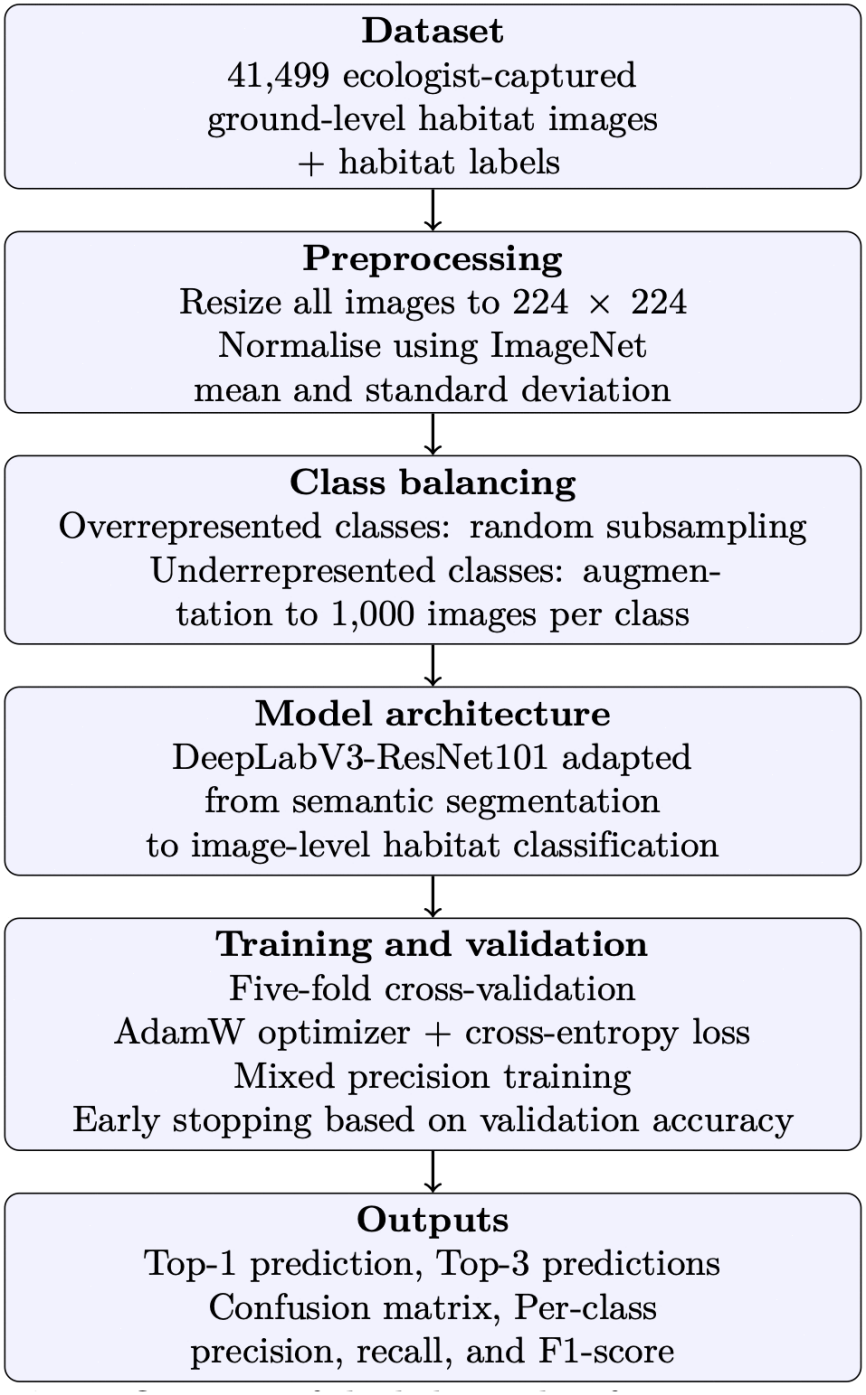
Overview of the habitat classification training and evaluation pipeline. Preprocessed and balanced habitat images were used to train a DeepLabV3-ResNet101 classifier, which was evaluated using five-fold cross-validation and standard classification metrics.

The validation images were transformed through a simpler transformation pipeline without augmentations to ensure consistent evaluation. This approach is standard in machine learning: data augmentations (e.g., random cropping, flipping, color jitter) are applied during training to improve generalisation by exposing the model to a wider variety of inputs. However, such augmentations are not applied to validation images because the goal during validation is to evaluate model performance on data that resembles real-world, unseen inputs as closely as possible. Applying augmentations during validation could introduce artificial variation and lead to misleading performance estimates. An overview of the habitat classification training and evaluation pipeline is shown in Fig 3.

### Evaluation metrics

Model performance was evaluated using several standard classification metrics: accuracy in Eq 1, precision in Eq 2, recall in Eq 3, and F1-score Eq 4, calculated on a per-class basis. Metrics were defined as follows:


$$\begin{array}{c} \text{Accuracy}=\frac{\text{Number of Correct Predictions}}{\text{Total Number of Predictions}}=\frac{TP+TN}{TP+TN+FP+FN} \end{array}$$ (1)

$$\begin{array}{c} \text{Precision}=\frac{TP}{TP+FP} \end{array}$$ (2)

$$\begin{array}{c} \text{Recall}=\frac{TP}{TP+FN} \end{array}$$ (3)

$$\begin{array}{c} \text{F1}=2\times \frac{\text{Precision}\times \text{Recall}}{\text{Precision}+\text{Recall}} \end{array}$$ (4)

where TP, TN, FP, and FN refer to true positives, true negatives, false positives, and false negatives, respectively. These values were calculated separately for each class using a one-vs-rest approach, where each class is treated as positive and all others as negative. Precision shows how many predicted positives were correct, recall shows how many actual positives were found, and the F1 Score combines both to give an overall measure of performance [32]. Confusion matrices were used to visualise misclassification patterns.

In addition, categorical cross-entropy loss was used to evaluate model performance on the validation set after each training epoch. This loss measures the difference between the predicted class probabilities and the true class labels. It is defined in Eq 5 as:


$$\begin{array}{c} ℒ=-\sum_{i=1}^{C}y_{i}\log({\hat{y}}_{i}) \end{array}$$ (5)

where *C* is the number of classes, *y*_*i*_ is the true label, and ${\hat{y}}_{i}$ is the predicted probability for class *i*. Validation loss is used both to monitor generalisation and as the criterion for early stopping.

To ensure stability, metrics were computed across cross-validation folds and aggregated for analysis. Furthermore, metadata for each test image across all folds was recorded, including the predicted and true labels, class probabilities, and the top-3 most likely habitat classes predicted by the model. This enriched metadata enables deeper analysis of model uncertainty, for example, instances where the correct label was not the top-1 prediction but still appeared within the top-3 candidates.

## Results

Baseline experiments using InceptionV3 and Inception–ResNet-v2 classifiers achieved peak top-1 accuracies of 0.44 and 0.55, respectively, with corresponding macro-F1 scores of 0.39 and 0.46. In contrast, the DeepLabV3–ResNet101 model achieved higher and more stable performance across folds (top-1 accuracy 0.59, macro-F1 0.61). A summary of the comparison is presented in Fig 2 Comparison of performance metrics indicates that the scene-oriented DeepLab architecture is better suited to this task than standard ImageNet-based classifiers, likely because it more effectively captures outdoor scene context and structural information across multiple spatial scales.

Also, as shown in Fig 2, a second standard classification model using InceptionV3 was initially tested, achieving a peak validation accuracy of 44%; its learning was unstable and prone to early convergence. The averaged precision, recall, and F1-score were 0.40, 0.38, and 0.39, respectively, reflecting a limited ability to generalize across habitat classes. Based on these limitations, DeepLabV3-ResNet101, a model originally designed for semantic segmentation but adapted here for image-level classification, was explored. The DeepLab-based model achieved significantly higher performance, with an average validation accuracy of 0.61, and averaged precision, recall, and F1-score of 0.63, 0.61, and 0.61, respectively. This improvement highlights the benefit of architectures that can better capture and use spatial information in an image for example, by looking at larger areas at once, combining features at different scales, or learning how different parts of the image relate to each other. This kind of model is especially useful for complex scene classification tasks like habitat recognition, where details such as vegetation patterns and background landscape provide important context.

**Fig 4:**
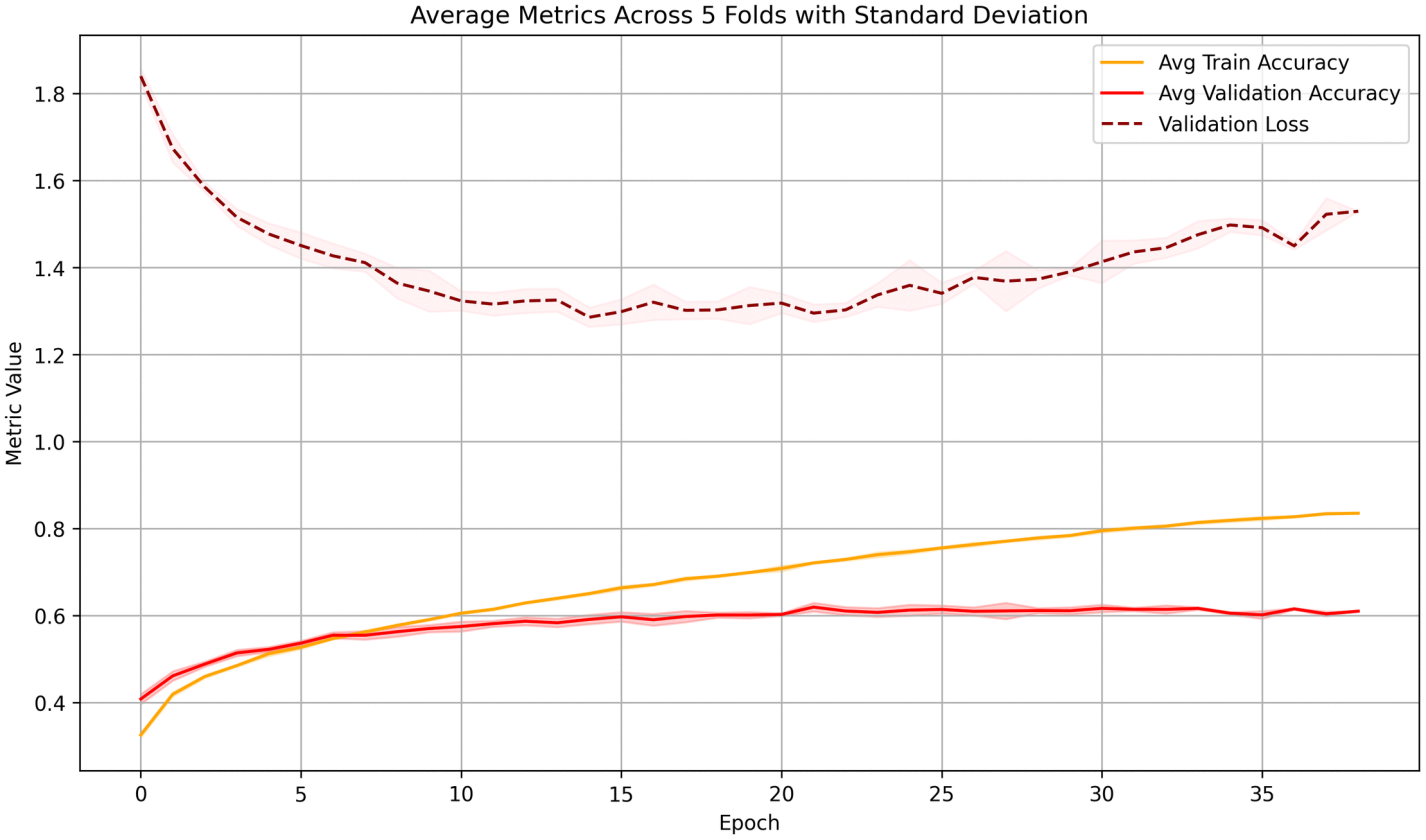
Average training accuracy, validation accuracy, and validation loss across 5-fold cross-validation, with shaded error bands representing the standard deviation at each epoch. Training accuracy steadily increases, while validation accuracy plateaus after around epoch 20, suggesting the onset of overfitting. Validation loss mirrors this trend, decreasing initially but rising again in later epochs. Early stopping occurred at epochs 29, 33, 30, 34, and 28 for folds 1 through 5, respectively.

The DeepLabV3-ResNet101 model demonstrated steady learning across folds, with validation accuracy ranging from approximately 60% to 63% before early stopping. Fig 4 shows the average accuracy metrics across all folds. The model achieved consistent improvements in training and validation accuracy during the initial epochs, with validation performance plateauing around 63%. While training accuracy continued to rise, suggesting some overfitting, early stopping was applied based on validation accuracy. Specifically, training was halted if validation performance did not improve for a fixed number of epochs (patience = 7), and the model parameters from the best-performing epoch were retained. This strategy helped mitigate overfitting and ensured that evaluation was based on the most generalizable version of the model.

Habitat classes can be broadly grouped into those defined primarily by visual structure (e.g., *Bare Sand* (BS), *Water* (WAT), *Coniferous Woodland* (CW), *Built up areas and Gardens* (BUAG), which achieved F1-scores above 0.85, and those defined primarily by floristic composition or subtle abiotic gradients (e.g., *Unimproved Grassland* (UG), *Fen, marsh and swamp* (FMS), *Dwarf Shrub Heath* (DSH), which showed consistently lower performance.

**Fig 5:**
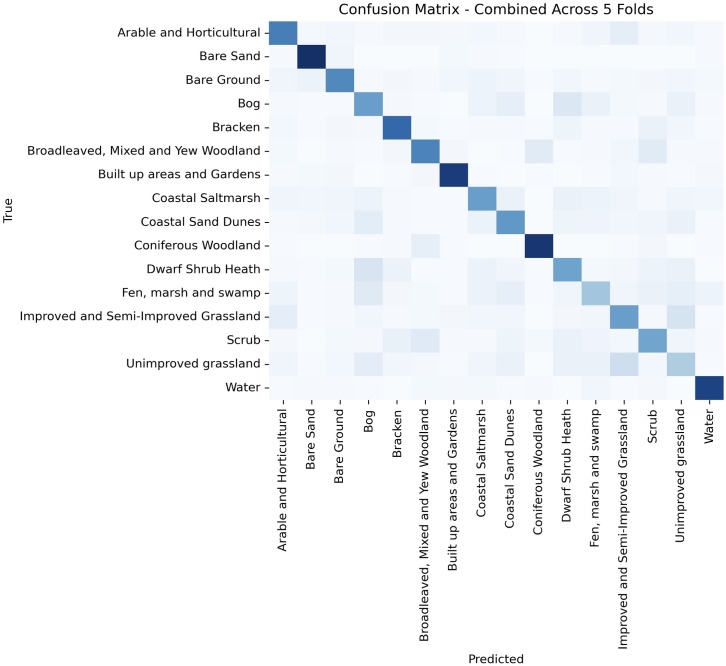
Confusion matrix showing class-wise prediction outcomes (summed across five folds). Diagonal values indicate correct predictions, while off-diagonal values show evidence of systematic prediction errors for some pairs of habitat types.

Table 2 summarises the average precision, recall, and F1-score for all 16 classes. Fig 5 shows classification accuracy and also reveals patterns of mis-classification. The per-class performance varied considerably.

**Table 2:** Per-class performance metrics averaged across 5 folds.

Class	Precision	Recall	F1-Score
Arable and Horticultural	0.68	0.62	0.64
Bare Ground	0.69	0.59	0.63
Bare Sand	0.85	0.90	0.87
Bog	0.49	0.54	0.51
Bracken	0.72	0.72	0.72
Broadleaved, Mixed and Yew Woodland	0.65	0.61	0.63
Built Up Areas and Gardens	0.87	0.86	0.86
Coastal Saltmarsh	0.60	0.52	0.56
Coastal Sand Dunes	0.56	0.56	0.56
Coniferous Woodland	0.83	0.92	0.87
Dwarf Shrub Heath	0.48	0.54	0.51
Fen, marsh and swamp	0.45	0.38	0.41
Improved and Semi-Improved Grassland	0.50	0.55	0.53
Scrub	0.52	0.49	0.50
Unimproved Grassland	0.37	0.34	0.35
Water	0.81	0.89	0.85

Inspection showed that classes with well-defined visual features and less intra-class variation (e.g., *Coniferous Woodland* (CW), *Bare Sand* (BS), *Built up areas and Gardens* (BUAG)) performed better, typically exhibiting strong diagonal dominance in confusion matrices and F1-scores above 0.85. In contrast, ambiguous or visually mixed classes such as *Unimproved Grassland* (UG) were often confused with others, as seen in their low recall and precision values. Examining classification performance more closely, even well-performing classes are subject to some misclassification.

**Fig 6:**
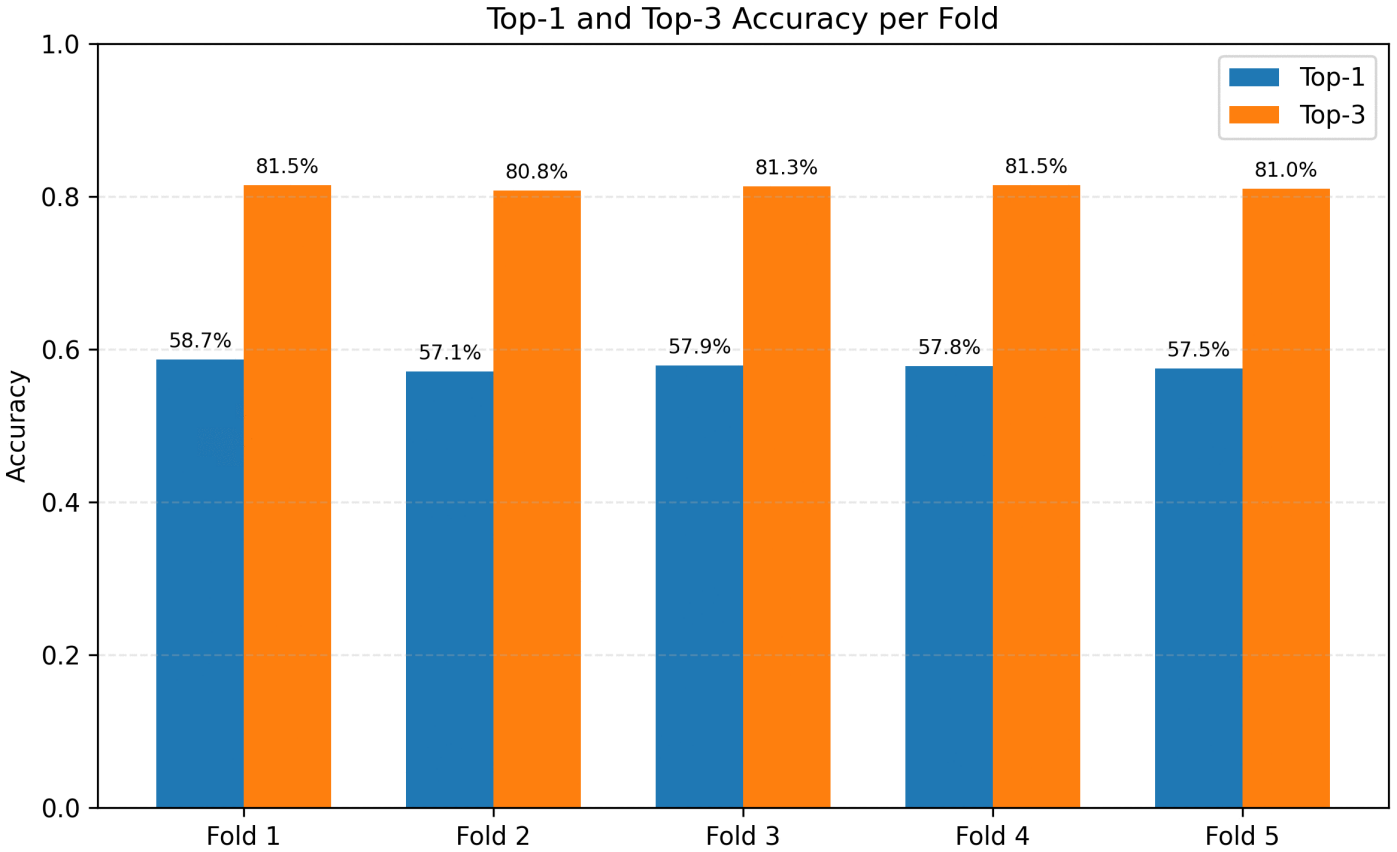
Top-1 vs. Top-3 classification accuracy across five cross-validation folds. While Top-1 accuracy remains consistent around 57- 59%, considering the Top-3 predictions significantly improves accuracy to approximately 80 - 82%, indicating the model often ranks the correct class among its top predictions.

To further understand model behaviour, stored metadata were analysed, including predicted and true labels, top-3 predictions, and confidence scores. Fig 6 visualises the frequency with which the true label appeared in the top-1 or top-3 predicted labels across the five cross-validation folds. The Top-1 accuracy reflects the proportion of predictions where the model’s first choice matches the true label. Top-3 accuracy indicates cases where the true label appears among the top three predicted classes, offering insight into the model’s performance when some uncertainty is allowed. The overall Top-1 classification accuracy across all five folds ranged from 57.0% to 59%, while Top-3 accuracy varied between 80% and 82%. This indicates that even when the Top-1 prediction was incorrect, the true label often appeared among the Top-3 predictions, especially for visually ambiguous classes such as *UG*. This suggests that Top-3 confidence rankings retain ecologically useful information and could support soft-label evaluation or interactive human-in-the-loop feedback systems.

Close examination of the confusion matrix in Fig 5 reveals several consistent patterns of misclassification across habitat classes. While most categories are accurately identified, the off-diagonal values highlight visually ambiguous habitat types that the model struggles to separate in ground-level photographs.

For instance, *Bare Ground* (BG) is most frequently confused with *Bare Sand* (BS) and *Coastal Sand Dunes* (CSD). This likely reflects the similar appearance of exposed sandy or soil-dominated surfaces in ground-level perspectives, where colour, texture, and lack of vegetation create limited visual cues for differentiation. *Water* (WAT), although generally distinctive, was occasionally misclassified as *Bare Sand* (BS) or *Coastal Saltmarsh* (CS). These errors may arise from reflective surfaces, wet substrates, or shoreline transitions that visually mimic shallow water or damp sediment in photographs.

**Fig 7:**
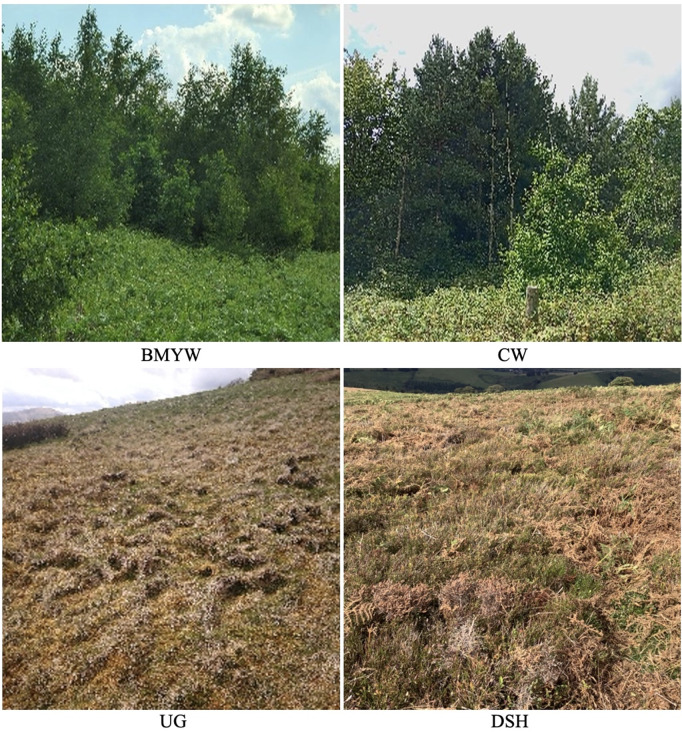
Examples of misclassifications due to visual similarity between habitat classes. **Top row:** An image from the *Broadleaved, Mixed and Yew Woodland* (BMYW) class (top-left) was misclassified as *Coniferous Woodland* (CW, top-right). Both exhibit dense green vegetation and overlapping structural features, likely contributing to the confusion. **Bottom row:** A *Unimproved Grassland* (UG) image (bottom-left) was predicted as *Dwarf Shrub Heath* (DSH, bottom-right). These habitats share similar visual elements, such as low vegetation and heterogeneous textures, which may explain the frequent misclassifications between them.

As shown in Fig 7, Confusion is also evident between *Broadleaved, Mixed and Yew Woodland* (BMYW) and *Coniferous Woodland* (CW), with each class occasionally predicted as the other. In ground-level images, dense tree cover, shadows, and partial occlusion can obscure differences in leaf shape or canopy form, making woodland types challenging to distinguish when photographed from within or at the edge of a stand. Scrub, which represents an intermediate vegetation structure between grassland and woodland, was also frequently predicted as Improved and Semi-Improved Grassland or Dwarf Shrub Heath. This is consistent with the visual overlap among low, bushy vegetation, mixed herbaceous layers, and short-statured shrubs commonly seen in these habitats. Some classes, particularly *Unimproved Grassland* (UG), show more diffuse confusion patterns. UG is often misidentified as *Dwarf Shrub Heath* (DSH) and *Scrub*, likely due to similarities in ground textures and fine-scale vegetation structures that appear visually alike in ground-level views. Likewise, categories with inherently mixed or heterogeneous features exhibit greater variability in their appearance, which contributes to lower precision and recall.

Overall, these patterns suggest that while many habitat types are reliably distinguished from ground-level imagery, visually ambiguous or transitional environments, those with shared vegetation structure, similar colour palettes, or variable composition, remain challenging.

A useful next step would be to compare model predictions with human classifications on the same test images. This would help determine whether errors arise from model limitations or from genuine visual ambiguity in the photographs.

These insights can guide future efforts in dataset refinement, targeted augmentation, and more discriminative modelling approaches for complex ecological classes.

These examples suggest that some classification errors might stem not from model weaknesses but from genuine ecological or visual ambiguities, underscoring the need for either additional metadata or multi-label classification approaches in future work.

**Fig 8:**
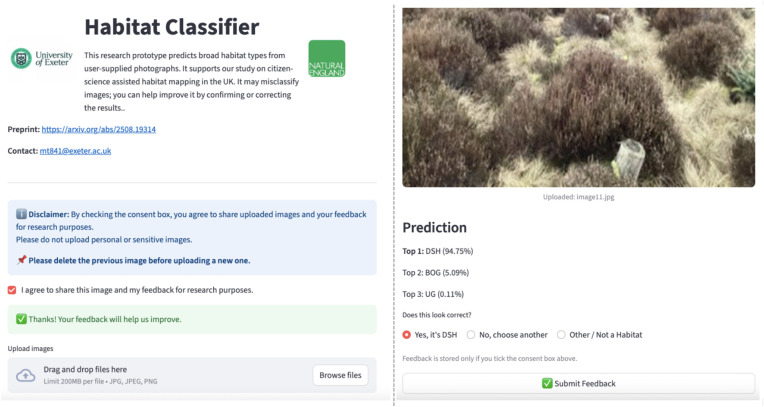
The landing page of the Habitat Classifier app, allowing users to upload ground-level habitat images for classification. An example of prediction results from the app showing top-3 habitat class predictions for an uploaded image.

To facilitate practical deployment and public engagement, a web-based application for habitat classification was developed. The app is available at Habitat Classification Web App and is built using Streamlit, a lightweight Python framework for interactive data apps. The interface allows users to upload one or more ground-level habitat images (in .jpg, .jpeg, or .png format), which are processed through a pre-trained DeepLabV3-ResNet101 classifier. Images are resized to 224×224 pixels and normalized using ImageNet statistics prior to inference. The model outputs the top-3 predicted habitat classes along with their associated probabilities and definitions. In addition to providing predictions, the updated application supports user feedback collection to improve future model iterations. Users are prompted to confirm or correct the predicted label, or to specify a custom label if the result is inaccurate or ambiguous. These interactions are logged, along with the uploaded image and prediction confidence, into structured .csv and .json files. With user consent, submitted images and annotations are saved locally to support downstream research and dataset expansion. The application also includes basic safeguards such as a consent checkbox and reminders not to upload personal or sensitive content. It serves as a prototype for a future mobile tool designed to support ecological monitoring in the field, targeting both professionals and citizen scientists. Screenshots of the interface are shown in Fig 8.

**Fig 9:**
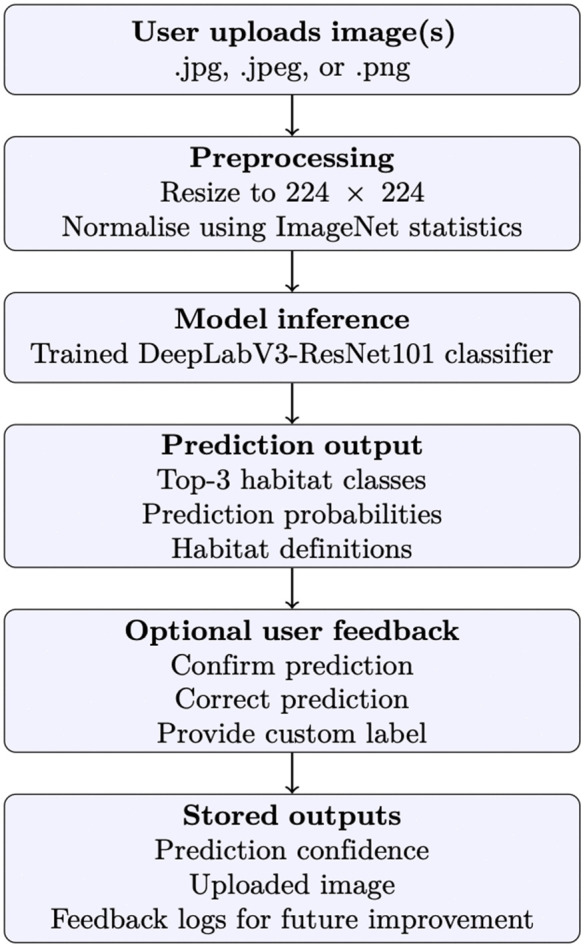
Overview of the web application inference workflow. Uploaded RGB habitat images are preprocessed and passed to the trained DeepLabV3-ResNet101 classifier, which returns the top-3 predicted habitat classes and associated probabilities. Optional user feedback can then be recorded for future model improvement.

The web application inference workflow is shown in Fig 9.

## Discussion

In this study, an automated visual classification system for identifying UK habitat types from ground-level photographs was developed. By deliberately restricting the analysis to ground-level visual information, this study isolates which components of a national habitat classification scheme are encoded in visual structure, and which depend on non-visual ecological criteria. Using a modified DeepLabV3-ResNet101 architecture and a balanced dataset of 16 habitat classes, the results demonstrate that accurate image-based classification is feasible even for complex ecological scenes. When seeking to predict a single correct habitat type for an image, the models achieved a mean F1-score of 0.63 across five-fold cross-validation, with high performance for visually distinct classes such as *Bare Sand* (BS), *Coniferous Woodland* (CW) and *Water* (WAT), and lower performance for visually mixed or ambiguous classes like *Unimproved Grassland* (UG). For a more relaxed task of identifying the correct habitat type within the top-3 most likely predicted labels, Top-3 accuracy increased to 0.81.

These findings indicate that fine-tuned convolutional neural networks (CNNs) can effectively classify terrestrial habitats from ecologist-captured, ground-level imagery. The models demonstrated consistent performance across most habitat categories, achieving moderate to high per-class accuracy using RGB data alone, particularly for classes with distinct visual characteristics. Classification outcomes varied by habitat type. Classes with strong visual features, such as *Water* bodies (WAT) or *Built up areas and Gardens* (BUAG), achieved the highest precision and recall, suggesting that CNNs are well suited to detecting visually distinct habitats. In contrast, ambiguous or transitional categories, such as *Unimproved Grassland* (UG), exhibited lower performance, with models frequently confusing them with neighboring classes. These challenges reflect broader issues in land cover classification, where visually similar habitats often produce overlapping features and annotation difficulties [22,23].

The patterns observed in this study align with findings from related domains. For example, wildlife image classification and marine habitat detection have similarly shown CNNs to be effective for structured ecological imagery [13,17]. However, like in those fields, performance drops were observed for classes with greater within-class variation or unclear boundaries. This reinforces known limitations of deep CNNs when applied to complex natural imagery without supporting metadata. Several constraints on this study might limit the generalisability of the results. Firstly, the model was trained exclusively on habitat classes defined within the Living England framework, and the training images were collected in England under a specific field protocol. Consequently, the classifier implicitly learns the visual and ecological boundaries adopted in this national scheme. Its performance outside England, or under alternative habitat taxonomies, is therefore unknown. Habitat classes used by other national or international mapping programmes may differ in both definition and spatial resolution, and the present model does not attempt to translate between classification systems.

A second limitation concerns the source of imagery. All photographs were taken by professional ecologists during structured field surveys, typically using consistent viewpoints, distances, and seasonal conditions. Such images differ systematically from photographs taken by members of the public, by citizen-science participants, or by automated field devices. The robustness of the classifier to variations in framing, lighting, device quality, and user behaviour has not been evaluated, and any deployment with images from other sources would require additional testing and validation.

Third, the study lacks an external test set from an independent survey campaign. Model evaluation relied on five-fold cross-validation within a single dataset, which provides a reliable estimate of performance on images drawn from the same distribution but does not assess performance on out-of-distribution conditions, such as new survey regions, rare habitat combinations, or unusual environmental contexts.

Finally, the model is constrained to predicting one of the 16 Living England habitat classes. It does not handle rare habitat types that fall outside this taxonomy, or cases where multiple habitat types co-occur in a single image. The approach also operates entirely on visual appearance, meaning that ecological factors central to habitat definitions, such as species composition, soil characteristics, and hydrological conditions, are inferred only through visual proxies. These factors limit the precision with which certain ecologically similar classes can be separated.

Together, these considerations indicate that applications of the classifier beyond its training conditions, including potential use in citizen-science or public-facing contexts, would require additional validation to confirm performance in the target conditions.

Misclassification patterns must be interpreted in ecological and practical terms, because the consequences of such errors differ substantially across habitat types and decision-making contexts [33,34]. However, habitat classes that are typically visually distinct in ground level photographs, such as *Bare Sand* (BS), *Water* (WAT), and *Coniferous Woodland* (CW), are typically classified and differentiated with high accuracy. These results thus offer valuable information to applications requiring coarse distinction between habitat classes, such as broad-scale screening, landscape reporting or exploratory survey planning.

The Top-3 accuracy of approximately 80−82% indicates that, even when the Top-1 prediction is incorrect, the model reliably narrows the correct habitat type to a small set of plausible candidates. This behaviour reflects the model’s ability to capture the general structural characteristics of habitats, even where fine-grained ecological distinctions are visually subtle or ambiguous in ground-level imagery. In many ecological workflows, a shortlist of likely habitat types is sufficient for decision support, enabling practitioners to combine automated suggestions with contextual knowledge, site metadata, or expert judgement [11,13]. This aligns with the broader observation that ground-level photographs often contain enough visual information to distinguish broad habitat structure, while more detailed ecological identification may require supplementary data or human interpretation.

However, for applications associated with regulatory decisions, land management payments, or formal habitat designation, misclassification can carry substantially greater ecological consequences. Confusion between *Unimproved Grassland* (UG), *Dwarf Shrub Heath* (DSH), and *Scrub* (SCR), for example, reflects genuine visual similarity in ground-level imagery but obscures important ecological differences. These habitat types differ in species composition, soil structure, and management requirements [1,2], and therefore cannot be substituted without potential risk to conservation outcomes. Similar issues arise for *Broadleaved, Mixed and Yew Woodland* (BMYW) versus *Coniferous Woodland* (CW), where differences in structure and species assemblages imply distinct ecological functions, restoration priorities, and carbon storage dynamics. In such cases, automated predictions should be interpreted cautiously and verified using additional data sources, contextual information, or expert ecological judgement.

Some misclassification errors may have more limited practical impact. Confusion between *Bare Sand* (BS) and *Coastal Sand Dunes* (CSD), for instance, often reflects real-world spatial adjacency and similar surface textures in ground-level photographs. The ecological distinction between these habitats frequently depends on geomorphological context, elevation, and vegetation cover [3], factors that may not be fully observable within a single image. Overall, the misclassification patterns observed in this study suggest that ground-level image classification primarily captures structural and visual appearance rather than the full set of biotic and abiotic factors used to define habitat types [18]. Accordingly, automated predictions are best integrated with contextual information such as location, topography, and species indicators where available.

An additional limitation of the current approach is that the model operates on full images and does not distinguish between habitat-relevant regions and potentially uninformative background content such as the sky. Sky regions may introduce variation associated with weather, illumination, and camera framing, which could reduce classification robustness for some habitat classes. However, sky visibility and horizon structure may also provide useful scene level information, especially in open habitats. Future work could therefore evaluate masking or segmentation based approaches that selectively retain ground and vegetation while excluding less informative image regions, and compare their performance against full image classification.

The role of foreground objects depends on the habitat class. In many cases, elements such as buildings or other human made structures may occlude vegetation and reduce the visibility of habitat defining features, potentially degrading classification performance. However, for certain classes such as *Built up areas and Gardens* (BUAG), the presence of buildings is itself a defining characteristic and provides useful information for classification. This dual role of foreground structures highlights a limitation of the current approach, which does not distinguish between informative and non informative occlusions. Future work could explore segmentation based or attention based methods to selectively retain relevant features while suppressing irrelevant foreground objects.

A key limitation of the image-only approach adopted here is the absence of explicit floristic information, which underpins most established habitat and vegetation classification systems. Vegetation ecology has long demonstrated that many habitat types are distinguished primarily by species assemblages rather than by overall structural appearance [5,6]. For example, grassland communities classified under the National Vegetation Classification (NVC) are separated by indicator species groups, soil chemistry, or management history, differences that may not be visually detectable in ground-level photographs. This likely contributes to the lower classification performance observed for classes such as *Unimproved Grassland* (UG) and other transitional habitats, where structural similarity masks substantial ecological differentiation.

The mismatch between floristic units and visual structure is well documented in vegetation ecology: habitats that share physiognomy can diverge markedly in species composition, ecological function, and management requirements. Similar challenges have been reported in satellite-based habitat mapping, where spectral or structural similarity often leads to confusion between floristically distinct communities. The results presented here are consistent with these findings, with habitat classes defined largely by plant species composition rather than dominant structural form exhibiting lower model performance.

Addressing these limitations may require integrating floristic proxies such as phenological signals, functional traits, or fine-scale texture features, alongside environmental metadata including soil properties, hydrology, and elevation, which traditionally inform vegetation survey interpretation [6]. Combining such ecological signals with image-based classifiers may allow future models to better reflect the ecological foundations of habitat classification frameworks.

The consistently high Top-3 accuracy observed in this study supports a practical hybrid workflow in which automated classification is used to generate a small set of candidate habitat types, with final selection made by a trained surveyor or a guided citizen-science participant. Such an approach balances efficiency with ecological validity, reduces cognitive load on human annotators, and preserves expert judgement for ambiguous or transitional habitats. Comparable human–machine workflows have proven effective in other areas of ecological monitoring, including camera-trap species identification and marine habitat assessment [13,17].

To improve classification for visually ambiguous classes, future work could explore integrating additional metadata, such as GPS coordinates, elevation, soil type, and seasonal indicators. These contextual cues may help disambiguate classes with overlapping visual characteristics and support more robust predictions across ecological gradients.

Model enhancements through architectural innovations also offer promising directions. Transformer-based vision models (e.g., Vision Transformers [35]) may improve classification by capturing global spatial dependencies and long-range contextual features [35]. This could be particularly useful for habitats with subtle texture differences across spatial scales. Ensemble learning approaches may also reduce variance and improve generalisation by combining complementary classifiers [36], especially when individual models overfit specific classes or data distributions. Few-shot learning methods may further aid underrepresented or difficult classes like *Multiple* by enabling models to learn from limited examples [37]. These techniques have already demonstrated success in biodiversity and medical imaging contexts.

Accurate habitat classification contributes to broader ecological goals, including biodiversity assessment, conservation planning, and environmental monitoring. As taxonomic standards and classification schemes vary across regions and institutions, developing models that can generalize or be translated between different habitat taxonomies is critical. Aligning model outputs with standard classification frameworks (e.g., EUNIS, CORINE, or Living England) would facilitate broader integration with existing ecological datasets and policy frameworks.

Finally, to improve accessibility and practical impact, a web-based application was deployed that allows users to upload habitat images and receive top-3 model predictions. This app serves as a prototype for an eventual mobile tool that can support real-time habitat classification in the field. The intention is that such a tool could be deployed as a mobile phone application, enabling users to capture habitat images and receive real-time habitat-type predictions, thereby increasing accessibility and supporting large-scale, crowd-sourced ecological monitoring efforts across diverse environments.

## Supporting information

S1 Fig Temporal distribution of available image-date metadata by habitat class. Rows correspond to the 16 habitat classes and columns to year-month periods. Cell colour indicates the number of images associated with each period. These metadata were used only to describe temporal coverage in the dataset and were not included as model inputs.
(TIF)

S2 Fig Seasonal distribution of available image-date metadata by habitat class. Rows correspond to the 16 habitat classes and columns to the four meteorological seasons. Cell colour indicates the number of images associated with each season. These metadata were used only to describe temporal coverage in the dataset and were not included as model inputs.
(TIF)

## References

[1] Rodwell JS. British Plant Communities. vol. 1–5. Cambridge University Press; 1991.

[2] Joint Nature Conservancy Committee (JNCC). Phase 1 Habitat Survey Handbook. Revised Phase 1 Handbook incorporating Field Manual. 2006. Available from: https://jncc.gov.uk/resource/phase-1-handbook/

[3] Natural England. Living England 2022–23: Technical User Guide (NERR141). Natural England; 2024. NERR141. Available from: https://publications.naturalengland.org.uk/publication/5260859937652736

[4] Braun-Blanquet J. Plant Sociology: The Study of Plant Communities. New York: McGraw-Hill; 1932.

[5] Westhoff V, van der Maarel E. The Braun-Blanquet Approach. The Hague: Dr W. Junk; 1973.

[6] Kent M. Vegetation description and data analysis: A practical approach. 2nd ed. Chichester: Wiley-Blackwell; 2012.

[7] Davies CE, Moss D, Hill MO. EUNIS Habitat Classification Revised 2004. Paris: European Topic Centre on Nature Protection and Biodiversity; 2004.

[8] Poole GC, Frissell CA, Ralph SC. In-stream habitat unit classification: Inadequacies for monitoring and some consequences for management. J Am Water Resour Assoc. 1997;33(4):879-896. doi: 10.1111/j.1752-1688.1997.tb04112.x

[9] Jongman RHG, Mücher CA, Bunce RGH, Lang M, Sepp K. A Review of Approaches for Automated Habitat Mapping and their Potential Added Value for Biodiversity Monitoring Projects. J Landsc Ecol. 2019;12(3):53–69. doi: 10.2478/jlecol-2019-0015

[10] Zhao X, Wang L, Zhang Y, Han X, Deveci M, Parmar M. A review of convolutional neural networks in computer vision. Artif Intell Rev. 2024;57(4). doi: 10.1007/s10462-024-10721-6

[11] Barta Z. Deep learning in terrestrial conservation biology. Biol Futur. 2023;74(4):359–67. doi: 10.1007/s42977-023-00200-4 38227170

[12] Perry GLW, Seidl R, Bellvé AM, Rammer W. An Outlook for Deep Learning in Ecosystem Science. Ecosystems. 2022;25(8):1700–18. doi: 10.1007/s10021-022-00789-y

[13] Miao Z, Gaynor KM, Wang J, Liu Z, Muellerklein O, Norouzzadeh MS, et al. Insights and approaches using deep learning to classify wildlife. Sci Rep. 2019;9(1):8137. doi: 10.1038/s41598-019-44565-w 31148564 PMC6544615

[14] Selvaraju RR, Cogswell M, Das A, Vedantam R, Parikh D, Batra D. Grad-CAM: Visual Explanations from Deep Networks via Gradient-Based Localization. In: 2017 IEEE International Conference on Computer Vision (ICCV). 2017. p. 618–26.

[15] Otsuka R, Yoshimura N, Tanigaki K, Koyama S, Mizutani Y, Yoda K, et al. Exploring deep learning techniques for wild animal behaviour classification using animal‐borne accelerometers. Methods Ecol Evol. 2024;15(4):716–31. doi: 10.1111/2041-210x.14294

[16] Bi X, Fu Y, Wang P, Zhang Y, Yang Z, Hou F, et al. Ecosystem health assessment based on deep learning in a mountain-basin system in Central Asia’s arid regions, China. Ecol Indic. 2024;160:110503. doi: 10.1016/j.ecolind.2024.112148

[17] Game CA, Thompson MB, Finlayson GD. Machine learning for non-experts: A more accessible and simpler approach to automatic benthic habitat classification. Ecol Inform. 2024;81:102619. doi: 10.1016/j.ecoinf.2024.102619

[18] Leblanc C, Bonnet P, Servajean M, Chytrý M, Aćić S, Argagnon O, et al. A deep‐learning framework for enhancing habitat identification based on species composition. Appl Veg Sci. 2024;27(3):e12802. doi: 10.1111/avsc.12802

[19] UK Centre for Ecology & Hydrology. Land Cover Map 2020 (LCM2020). 2021;1. https://catalogue.ceh.ac.uk/documents/14a9ec05-071a-43a5-a142-e6894f3d6f9d

[20] Shi H, Norton L, Ridding L, Rolph S, August T, Wood CM, et al. Habitat classification from ground-level imagery using deep neural networks. arXiv preprint. 2025. doi: 10.48550/arXiv.2507.04017

[21] Mozaic Earth. Mozaic Earth: Ground-truth your nature data, at scale. 2025. https://www.mozaic.earth/

[22] Bothmann L, Wimmer L, Charrakh O, Weber T, Edelhoff H, Peters W, et al. Automated wildlife image classification: An active learning tool for ecological applications. Ecol Inform. 2023;77:102231. doi: 10.1016/j.ecoinf.2023.102231

[23] Mainali K, Evans M, Saavedra D, Mills E, Madsen B, Minnemeyer S. Convolutional neural network for high-resolution wetland mapping with open data: Variable selection and the challenges of a generalizable model. Sci Total Environ. 2023;861:160622. doi: 10.1016/j.scitotenv.2022.160622 36462655

[24] Kilcoyne AM, Clement M, Moore C, Picton Phillipps GP, Keane R, Woodget A, et al. Living England: Technical User Guide. York, UK: Natural England; 2022. https://publications.naturalengland.org.uk/publication/4918342350798848

[25] Natural England. Living England 2022–23: National-Scale Habitat Map for England. 2024. https://www.data.gov.uk/dataset/3a87c13c-1303-4452-9da4-5bb0ce84667f/living-england-2022-23

[26] Woodget A, Trippier P, et al. NERR141: Living England 2022–23 Technical User Guide. Natural England; 2024. NERR141. Available from: https://publications.naturalengland.org.uk/publication/5260859937652736

[27] Chen LC, Papandreou G, Kokkinos I, Murphy K, Yuille AL. Rethinking atrous convolution for semantic image segmentation. arXiv preprint arXiv:170605587. 2017. 10.1109/TPAMI.2017.2699184 28463186

[28] Deng J, Dong W, Socher R, Li LJ, Li K, Fei-Fei L. ImageNet: A large-scale hierarchical image database. In: 2009 IEEE Conference on Computer Vision and Pattern Recognition. IEEE; 2009. p. 248–55.

[29] Cubuk ED, Zoph B, Mané D, Vasudevan V, Le QV. AutoAugment: Learning Augmentation Strategies From Data. In: 2019 IEEE/CVF Conference on Computer Vision and Pattern Recognition (CVPR). 2019. p. 113–23.

[30] Micikevicius P, Narang S, Alben J, Diamos G, Elsen E, Garcia D, et al. Mixed Precision Training. arXiv preprint arXiv:171003740. 2018.

[31] PyTorch Core Team. Automatic Mixed Precision (AMP). 2020. https://pytorch.org/docs/stable/amp.html

[32] Sokolova M, Lapalme G. A systematic analysis of performance measures for classification tasks. Inf Process Manag. 2009;45(4):427–37. doi: 10.1016/j.ipm.2009.03.002

[33] Guisan A, Thuiller W, Zimmermann NE. Predictive habitat distribution models in ecology. Encyclopedia of Biodiversity. 2013. p. 276–88.

[34] Lindenmayer DB, Likens GE. Effective ecological monitoring. 2nd ed. Clayton, Australia: CSIRO Publishing; 2018.

[35] Dosovitskiy A, Beyer L, Kolesnikov A, Weissenborn D, Zhai X, Unterthiner T, et al. An Image is Worth 16x16 Words: Transformers for Image Recognition at Scale. arXiv preprint arXiv:201011929. 2020.

[36] Zhou ZH. Ensemble Methods: Foundations and Algorithms. CRC Press; 2012.

[37] Wang Y, Yao Q, Kwok JT, Ni LM. Generalizing from a few examples: A survey on few-shot learning. ACM Comput Surv. 2020;53(3):1–34.

